# Complete Heart Block in Thyrotoxicosis, Is It a Manifestation of Thyroid Storm? A Case Report and Review of the Literature

**DOI:** 10.1155/2012/318398

**Published:** 2012-08-11

**Authors:** Rashed Al Bannay, Aysha Husain, Saeed Khalaf

**Affiliations:** ^1^Cardiology Unit, Internal Medicine Department, Salmaniya Medical Complex, Manama, Bahrain; ^2^Endocrinology Unit, Internal Medicine Department, Salmaniya Medical Complex, Manama, Bahrain

## Abstract

Thyrotoxicosis complicated by advance degree atrioventricular block, a rare complication of a common disease. The term apathetic thyrotoxicosis, where palpitations and cardiac involvement are the sole manifestations of disease, is well known. Thyroxin's ability to sensitize the catecholamine receptors causing tachyarrhythmias is well addressed. However, as an aetiology for advanced heart block, thyrotoxicosis is ranked as one of the rarest.

## 1. Case Presentation

Mrs. T.R.M. is a 37-yr-old Egyptian woman who presented with a history of shortness of breath, palpitations, and recurrent syncope that had lasted three days.

The patient has been thyrotoxic for the last 10 years with erratic follow-up appointments and poor adherence to her treatment program.

At presentation, she had progressive shortness of breath that had been occurring for the past 3 days and was associated with palpitations that she perceived as slow and heavy.

She reported recurrent, unprovoked syncopal attacks that coincided with her palpitations. She had no history of chest pain or ankle oedema.

In a review of systems, she reported diarrhoea associated with colicky abdominal pain and fever.

The patient is a housewife who has had six pregnancies, the most recent of which was in 2008. The mode of delivery was a lower segment Cesarean section due to a twin pregnancy that had been complicated by placenta previa and antepartum hemorrhage.

The remainder of her obstetric history was significant, including two abortions and two intrauterine deaths. Her first pregnancy was uneventful.

She justified her noncompliance with antithyroid medications by relating these drugs to her complicated obstetric history.

On examination, she was apprehensive, restless, mildly icteric, and had a low-grade fever.

Her blood pressure was 140/80 mmHg, and she had a heart rate of 54 beats/min.

Her extremities were moist, and she was sweating excessively. 

She had a diffuse goitre with bruit. Thyrotoxicosis-related eye signs, such as lid retraction and lid lag, were evident.

Cannon A waves were visible in her jugular venous pressure (JVP). 

Auscultation of the precordium demonstrated audible heart sounds with variable S1 intensity.

Her lungs were clear, and she was not experiencing congestive heart failure.

The abdominal examination was unremarkable except for diffuse, mild tenderness, and active bowel sounds.

The basic metabolic workup is shown in the Table 1.

The septic workup and autoimmune profile were normal.

**Figure 1:**
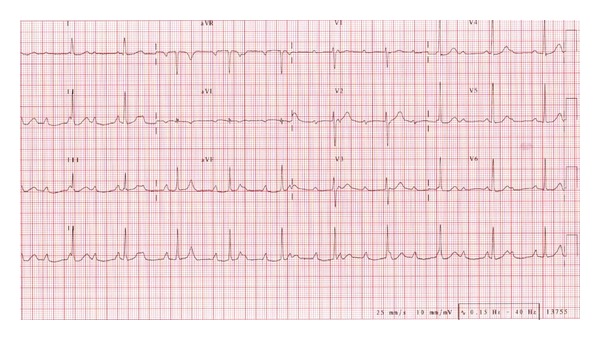
Electrocardiogram showing advance degree atrioventricular block at time of admission.

Her electrocardiogram showed complete heart block with narrow QRS complexes (Figure 1).

The echocardiogram showed preserved left ventricular systolic function with no valvular lesions.

She was admitted to the cardiac care unit for closer monitoring. She consented to transvenous temporary pacing in the event that it was required.

Antithyroid medications, in the form of propylthiouracil (PTU) with parenteral steroid therapy, were initiated.

**Figure 2:**
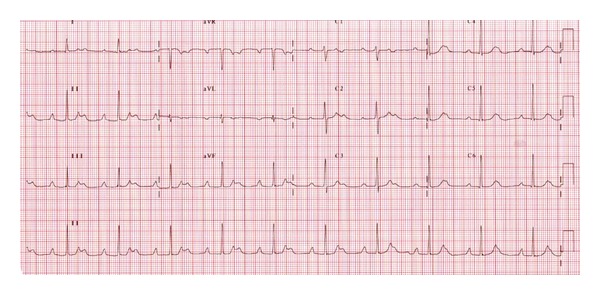
Electrocardiogram showing second-degree heart block performed on third day of anithyroid medication.

**Figure 3:**
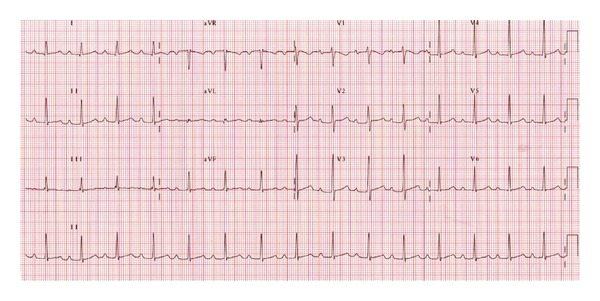
Electrocardiogram showing first-degree heart block performed on fifth day of antithyroid medication.

The patient remained hemodynamically stable, and by the third day of antithyroid medication, her heart rhythm transited into second degree AV block (Figure 2); by the fifth day of medication, she was in first-degree atrioventricular block AV block (Figure 3).

She was discharged seven days after admission. Her antithyroid medications were upgraded, and the steroids were tapered.

**Figure 4:**
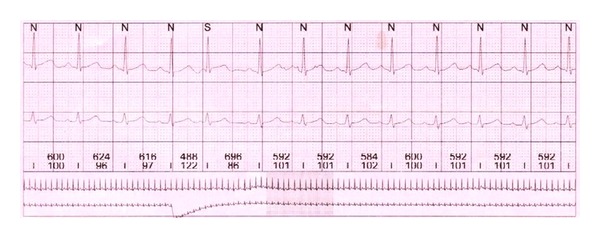
Holter rhythm strip showing normal sinus rhythm.

At the follow-up appointment, her holter monitor showed normal sinus rhythms with physiological heart rate variability, and she was in an euthyroid state (Figure 4).

Due to her nonadherence to medications, she was offered iodine ablation of her thyroid, which she adamantly resisted.

The patient continued to figure prescribed medications and remained euthyroid for two months.

## 2. Discussion

Thyrotoxicosis is known to cause tachyarrhythmia, the leading example of which is atrial fibrillation [1]. A hyperthyroid status complicated by high-grade atrioventricular block (AV) has been well documented in the literature [2–10]. Its rarity renders it more difficult to recognize in daily practice. Because the treatment of hyperthyroidism includes a betablocking agent, recognizing high-grade atrioventricular block is critical before subjecting patients with concomitant bradyarrhythmia to the hazardous effects of betablockers. Syncopal attacks are an indication of such a diagnosis in the context of thyrotoxicosis [2, 3, 5, 10].

The collective evidence from the sporadic reported cases suggests that any part of the cardiac conduction system is vulnerable to the effects of high thyroxin [11–13].

The effects of high thyroxin can manifest as sick sinus syndrome, sinoatrial block, or various degrees of AV block [11–13].

The data in the literature that address the pathogenesis of high-grade AV block in the context of hyperthyroidism are primarily speculative. The positive chronotropic effect of thyroxin is well known; however, its negative dromotropic action is not widely recognized. The presence of interstitial inflammation in the AV node, the His-bundle and its branches, which are associated with the typical ECG changes in hyperthyroidism, is well recognized [9].

Focal inflammation of the myocardium is reported in patients subjected to high levels of thyroxin [14].

In our patient, the collective thyrotoxic signs and symptoms indicated the possibility of an impending thyroid storm. Ho et al. have previously reported the coexistence of thyroid storm with high-grade AV block [7]. In other words, a high-grade AV block can be a manifestation of severe thyrotoxicosis. Despite the sporadic existence of similar cases in the literature, at least 5 of the reviewed cases were either frank or impending thyroid storms [3, 6–9].

It may be worth considering high-grade AV block, which is a life-threatening emergency, to be one of the cardiovascular markers of a thyroid storm [15].

However, it has to be emphasized that in such a scenario, the treatment is identical to a severe thyrotoxic crisis, with the exception of avoiding beta-blocking agents. The mainstays of treatment are antithyroid medication with steroid therapy and monitoring the cardiac rhythm by telemetry. Temporary pacing is rarely indicated unless there is hemodynamic instability, and cardiac electrophysiology has no role in acute management [3, 6].

For cases in which the hyperthyroidism is intractable or the patient is poor complaint, thyroid iodine ablation or subtotal thyroidectomy are long-term alternatives [16].

## Figures and Tables

**Table 1:** Results of blood investigations done during patient's admission.

Laboratory investigation	Value	Reference range
White cell count	9.9 × 10^9^	3.6–9.6 × 10^9^/L
Hemoglobin	11.4 g/dL	12–14.5 g/dL
Platelets	431 × 10^9^	140–440 × 10^9^/L
Random blood sugar	7.1 mmol/L	3.6–8.9 mmol/L
Urea	3.6 mmol/L	3–7 umol/L
Creatinine	57 umol/L	53–118 umol/L
Calcium	2.44 mmol/L	2.13–2.63 mmol/L
Magnesium	0.79 mmol/L	0.74–1 mmol/L
Phosphorous	1.0 mmol/L	0.8–1.4 mmol/L
Sodium	135 mmol/L	140–148 mmol/L
Chloride	101 mmol/L	100–107 mmol/L
Potassium	4.8 mmol/L	3.6–5.2 mmol/L
Bicarbonate	23 mmol/L	21–30 mmol/L
Creatinine kinase	21 mmol/L	<67 u/L
Albumin	32 g/L	38–50 g/L
Globulin	50 g/L	15–30 g/L
Total bilirubin	56 umol/L	<18 umol/L
Alkaline phosphatase	102 u/L	50–135 u/L
Alanine aminotransferase	57 u/L	30–65 u/L
g-glutamyltransferase	32 u/L	5–85 u/L
ESR	60	0–20 mm/hr
Thyroid stimulation hormone	0.0 uIU/mL	0.25–5 uu/mL
Free T3	23.0 pmol/L	2.5–7.8 pmol/L
Free T4	94.6 pmol/L	6–24.5 pmol/L
Thyroglobulin	16.3 ug/L	3–42 ug/L
